# Differences in Presentation and Management of Pediatric Facial Lacerations by Type of Health Insurance

**DOI:** 10.5811/westjem.2015.4.25009

**Published:** 2015-07-02

**Authors:** Siraj Amanullah, James G. Linakis, Patrick M. Vivier, Emily Clarke-Pearson, Dale W. Steele

**Affiliations:** * Alpert Medical School of Brown University, Department of Emergency Medicine, Providence, Rhode Island; † Alpert Medical School of Brown University, Department of Pediatrics, Providence, Rhode Island; ‡ School of Public Health at Brown University, Providence, Rhode Island; § Harvard Medical School, Department of Plastic Surgery, Cambridge, Massachusetts

## Abstract

**Introduction:**

Limited data are available regarding differences in presentation and management of pediatric emergency department (PED) patients based on insurance status. The objective of the study was to assess the difference in management of pediatric facial lacerations based on medical insurance status.

**Methods:**

We conducted a retrospective cohort study with universal sampling of patients with facial lacerations who were treated in an urban PED (45K visits/year) over a one-year period. Demographic features and injury characteristics for patients with commercial (private) insurance and those with Medicaid or Medicare (public) insurance were compared.

**Results:**

Of 1235 children included in the study, 667 (54%) had private insurance and 485 (39%) had public insurance. The two groups did not differ in age or gender, arrival by ambulance, location of injury occurrence, mechanism of injury, part of face involved, length or depth of laceration, use of local anesthetic, or method of repair but differed in acuity assigned at triage. Patients with public insurance were found less likely to have subspecialty consultation in bivariable (OR=0.41, 95% CI [0.24–0.68]) and multivariable logistic regression analyses (OR=0.45, 95% CI [0.25–0.78]). Patients with public insurance received procedural sedation significantly less often than those with private insurance (OR=0.48, 95% CI [0.29–0.76]). This difference was not substantiated in multivariable models (OR=0.74, 95% CI [0.40–1.31]).

**Conclusion:**

Patients with public insurance received less subspecialty consultation compared to privately insured patients despite a similarity in the presentation and characteristics of their facial lacerations. The reasons for these disparities require further investigation.

## INTRODUCTION

Disparities in healthcare based on race and ethnicity have been investigated in a number of studies focusing on management of pain, emergency department (ED) triage and waiting time, screening for sexually transmitted diseases, diabetes, asthma, oral and dental care, cardiac care, dialysis, juvenile rheumatoid arthritis, cancer management and orthopedics care among others.^1^–^8^ However, limited data are available addressing disparities in emergency care for pediatric patients based on the patients’ insurance status.^9^–^13^ It is important to assess whether insurance-based differences in presentation and management exist in order to identify areas for quality improvement.^14^–^21^ While studies have demonstrated the limited availability of care for publicly insured patients and the diverse reasons for use of the ED by these patients, there have been few reports investigating differences in medical management based on the patient’s insurance status.^8^,^16^,^22^–^24^

The overall goal of this study was to identify whether there are disparities in the ED management of patients based on their insurance status. To optimally assess whether there is a difference in the ED management of a specific complaint, such a complaint would ideally require use of the ED irrespective of insurance status and should be a relatively common reason for utilization of the ED. Facial laceration was identified as a specific complaint to address this goal since pediatric patients with this diagnosis frequently require care in an emergency or urgent care setting rather than from their primary care physician.^25^ Therefore, this study was specifically designed to assess the differences in management of pediatric facial laceration based on medical insurance status. Management aspects hypothesized, *a priori*, to be different between the two groups were sub-specialty consultation and use of procedural conscious sedation.

## METHODS

### Study design, setting and population

We conducted a retrospective cohort study of visits at an urban children’s hospital pediatric emergency department (PED) and Level I trauma center with approximately 45,000 visits a year. We selected a universal sample of all patients presenting with facial lacerations from September 1, 2005, through August 31, 2006. Patients were identified based on discharge diagnosis through ICD-9 codes (870–873, and sub-categories under these codes). We performed a detailed chart review of computerized, scanned records for predetermined study variables.

### Measurements and study protocol

Study variables included age, gender, total time of visit, triage level (based on the Emergency Severity Index (ESI) triage system, further described below), mode of arrival (ambulance or by private transportation), laceration characteristics (depth and length), specialty consultation obtained (yes/no), use of procedural sedation (yes/no), and management of laceration (physician working in the ED vs. consulting physician performing repair, use of local analgesia, method of repair).

Insurance status was extracted from a demographic sheet which is available in each patient’s chart. We divided patients into two groups based on their insurance status, i.e., a private insurance group and a public insurance group. Public insurance patients were those with state Medicaid managed care program, Medicaid fee for service, or Medicare, as their expected source of payment. The private insurance group included all other commercial insurance groups (Blue Cross Blue Shield, Tufts, Aetna, etc). We excluded from the analysis those with unknown insurance status since, due to the retrospective nature of the data, their actual insurance status could not be verified.

Although data pertaining to patient race and ethnicity are typically recorded with the patient’s data, this information is often of uncertain accuracy, as reported in a number of studies.^26^,^27^ In our data set, approximately 24% of the race/ethnicity variable data was missing and it was uncertain if the available race/ethnicity information in the electronic record was reliable. Therefore, we decided not to include race and ethnicity data in the final analysis.

Location where the injury occurred, mechanism of injury, and intent of injury were coded by research assistants based on the National Electronic Injury Surveillance System All Injury Program data coding schema.^28^ As a measure of injury severity, an acuity level is routinely assigned when the patient presents to the ED, and is based on the five-level ESI triage system created by the Agency for Healthcare Research and Quality.^29^ We collapsed this acuity index into three categories: high (ESI 1 and 2), moderate (ESI 3) and low acuity triage levels (ESI 4 and 5). Triage categories were collapsed to allow for an adequate number of patients in each of the categories. For example, there were only three patients categorized as ESI 1 (none of which required sedation or had specialty consultation obtained for laceration repair).

Data pertaining to wound characteristics were extracted from the procedure notes and hence dependent on the recorded information. Wound description included location of injury on face, depth of the wound (superficial if not including the subcutaneous tissue), and length of the laceration (divided into three categories, less than 1cm, 1cm to 2cm, and greater than 2cm). We defined procedural sedation as use of intravenous medications at dosages to induce moderate or deep sedation and not for anxiolysis. In our ED, these included combination use of intravenous midazolam and ketamine.

The chart was reviewed to determine whether the laceration repair was performed by a physician based in the PED or by a specialty consultant. However, due to the retrospective nature of the study, data were lacking on the level of training of the physician performing the laceration repair. Physicians based in the PED could be an attending physician, a PED fellow, resident (emergency medicine, pediatrics, family practice), or a nurse practitioner. The consulting physician is typically a resident from either the plastic surgery or otorhinolaryngology (ENT) service and may be a resident training in the specific sub-specialty or a rotating resident from another sub-specialty, such as general surgery or emergency medicine, who is receiving subspecialty training in plastic surgery. Of note, in the PED every patient is evaluated directly by a PED attending physician who oversees (or, at times, performs) the laceration repair.

### Institutional Review Board Status and Statistical Analysis

This study was approved by the local institutional review committee. We calculated sample size to detect a difference of 10% in use of conscious sedation and specialty consultation between the two groups. For an alpha value of 5% and a power of 80%, at least 140 patients were required in each group. We decided to perform universal sampling for a duration of one year to oversample, thereby accounting for the possible limitation of missing information inherent to the retrospective nature of data collection. Data extraction was performed by a single trained research assistant who was not aware of the specific study question. The supervision of data collection and entry was conducted by the research coordinator. We analyzed data with SAS version 9.1.3 (SAS Institute, Inc, Cary, NC) and R.^30^ Chi-square tests of independence and Mann-Whitney tests were used for bivariable models where appropriate. We employed logistic regression models to adjust for demographic and wound characteristic covariates that might predict specialty consultation and use of conscious sedation. Patients who had a scalp laceration were excluded from the multivariable analysis as these patients are very unlikely to have either specialty consultation or conscious sedation. We included self–pay patients (who comprised 7% percentage of all patients with facial lacerations) as a separate category in the multivariable regression analysis, thereby providing a model that was more robust.

## RESULTS

For the study time period, 1,516 patients with facial lacerations were identified. Of these, we included 1,235 in the study after exclusion of patients with scalp lacerations (n=281). Six hundred sixty-seven (54%) had private insurance, 485 (39%) had public insurance, and 83 (7%) patients had no insurance documented at the time of services provided (considered to be self-pay). When comparing private and public insurance patients, median age and gender were similar (Table 1). The two groups also did not differ with respect to location where the injury occurred, the mechanism or intent of injury, or the mode of arrival at the hospital (Table 1). A significant difference was noted between the two insurance groups with respect to the triage level, with publicly insured patients more frequently assigned to the low acuity group (Table 1). In contrast, when laceration characteristics were assessed, no difference was found between groups in the following: part of the face affected, length, or depth of laceration (Table 1).

Twenty-one patients (4%) in the public insurance group had specialty consultation compared to 66 patients (10%) in the private insurance group, with bivariable analysis demonstrating that children with public insurance received significantly less specialty consultation (OR=0.41, 95% CI [0.24–0.68], p=0.0015). Similarly, there was a difference between groups in the physician performing the repair, with a specialty consultation team member repairing lacerations less often in the publicly insured group (p=0.04). Comparable results were noted for use of procedural sedation, with less use for public insurance patients (OR=0.48, 95% CI [0.29–0.76, p=0.007) than for those with private insurance (public insurance patients 5% [n=25], compared to private insurance patients 10% [n=68]; (Table 2). There was no difference in the total time spent in the ED for the two groups (Table 2).

We used a multivariable logistic regression analysis adjusted for age, gender, depth and length of laceration, and acuity to evaluate the effect of insurance status as a predictor of subspecialty consultation (Table 3). In the model assessing specialty consultation obtained, the public insurance group was less likely to have specialty consultation compared to the private insurance group (OR=0.45, 95% CI [0.25–0.78]). Specialty consultation was more likely to be obtained for female patients, those with deep or complex laceration, and those with high acuity assigned. For example, our model predicts that a two-year-old female patient with a superficial, 1–2cm long laceration, and intermediate acuity, had a 10% chance of specialty consultation if she had private insurance, compared to a 5% chance if she had public insurance.

We used a second model, adjusted for age, gender, specialty consultation, depth and length of laceration, acuity, and allowing for possible interactions between age and consultation, and between gender and consultation, to test the relationship between insurance status and use of procedural sedation (Table 3). In the adjusted model, the difference between private and public insurance was not found to be significant (OR=0.74; 95% CI [0.40–1.31]). Deeper lacerations or those with high acuity were found to be important variables for use of procedural sedation. The most important factor associated with the use of procedural sedation was specialty consultation (Table 3). The child in our example of a two-year-old female with superficial, 1–2cm long laceration, and intermediate acuity, the model would predict a 70% chance of procedural sedation use if a specialty consultation was obtained, compared to a 12% chance if specialty consultation was not obtained.

## DISCUSSION

The purpose of this study was to identify differences in care of children presenting to the ED with facial lacerations based on medical insurance status. After adjusting for age, gender, wound characteristics and acuity, we found a modest but significant association between private insurance and use of specialty consultation. Since the baseline presenting characteristics of the two groups and their injuries were similar, it is unlikely that the differences in care can be attributed to differences in types of injuries sustained. These results are in contrast to those of another study that examined race and socio-economic levels as possible drivers of disparity in use of sedation and anxiolysis for laceration repair of pediatric patients and documented no difference.^31^

The significant difference in specialty consultation for patients in the two insurance groups is noteworthy. The ED studied is a Level I trauma center and is continuously staffed by pediatric emergency physicians who have received training in the repair of facial lacerations and are qualified to perform the majority of repairs that present to the pediatric ED. A decision to request specialty consultation, when made by the PED attending, may be based on a variety of factors, including characteristics of the wound, and at times, parental request, but is not thought to be driven by insurance status of the patient. When specialty consultation is obtained, the repair is most commonly performed by a resident in plastic surgery or ENT surgery. Of note, it is rare to have an attending ENT or plastic surgery physician perform a repair in the PED. It is important to note that in the study population, if specialty consultation was obtained, then the patient was extremely likely to have received procedural sedation. This points out an important aspect of ED resource utilization driven by specialty consultation.

Studies have shown that publicly insured or uninsured patients have less access to specialist care.^9^ It is possible that the increased frequency of specialty consultation for the private insurance group in our study may have been driven by parental request. For example, the between-group difference noted in our study may be attributable to a lack of some parents’ healthcare knowledge regarding the possibilities for specialty care of facial lacerations, and hence less frequent requests by publicly insured parents to obtain a “plastics consult.”^32^ However, we cannot rule out subtle biases on the health caregivers’ part. The role played by these biases cannot be quantified, but conceivably could be the reason for differences between public and private insurance patients seen in this study. It is also noteworthy that when we controlled for specialty team consultation in the regression models, the use of conscious sedation was still greater in the private insurance group. This again may have been driven by parental requests for procedural sedation, although we do not have data specifically addressing this issue.

We also found a difference in the level of acuity assigned to patients in the two groups, with the private insurance group more likely to be assigned a moderate level of acuity and the public insurance group more likely to be assigned a lower level of acuity. This is, most likely, the result of the inter-relationship of a variety factors. Since, in our study, arrival via EMS was the same for the two groups, differences in acuity level assignment are unlikely to be attributable to arrival mode. One possibility is that acuity assigned may have been driven by parental request at triage for repair by a plastic surgeon. Since the ESI triage system is partially based on an estimation of anticipated resource utilization, an assessment by the triage nurse of need for subspecialty consultation or increased likelihood of procedural sedation may have resulted in a higher acuity level assignment in these patients. However, our data do not permit us to evaluate these possibilities. It is important to note, though, that in the multivariable model, including the acuity variable in the models did not change the findings related to use of specialty consultation and public insurance.

## LIMITATIONS

The retrospective study design and use of electronic records employed here have inherent limitations. Additionally, this study cannot address the various subjective and interactive factors guiding the disparity in care based on insurance status. Further, differential documentation of facial laceration by physicians could potentially introduce bias in the study. Because of the retrospective nature of this study, we were unable to ascertain whether the requests for surgical consultation originated from the parent or from ED staff. Similarly, we were unable to determine if the request for procedural sedation was driven by the parent or the surgical consultant. As noted, procedural sedation was highly likely to be used if specialty consultation is obtained. Many factors contribute to the decision to use procedural sedation, including both objective factors (laceration characteristics and age of patient), and other more subjective factors such as parental and provider perception of pain and provider comfort level for a specific procedure. There was limitation in identification of the level of training of the physician performing the procedure or the manpower available at the time of the repair, which may have guided the need to request assistance from the specialty resident. It seems possible that the level of training of the treating physician and comfort level based on clinical experience may have contributed to the decision to use sedation. Although there appears to be no reason to expect that there would be differences in this variable between physicians caring for children in the two insurance groups. Our data indicate that the two groups were equivalent with reference to the objective factors, although we were limited in our ability to quantify subjective features. Also, use of conscious sedation or repair by a specialty consultant does not necessarily translate into a better quality of care.

A lack of reliable data on the race and ethnicity of patients limits our ability to determine the extent to which these were confounding factors. Race and ethnicity have been found to be associated with disparities in care in several studies and are likely confounded with insurance status. Since we could not reliably document the exact race/ethnicity of the patients in our sample, we were unable to determine the extent to which they contributed to the disparities noted in our study. Of note, Brodzinski et al. noted no differences related to race in the use of procedural anxiolysis for laceration repair.^31^ The racial/ethnic distribution for the state where the study was conducted is 82% Caucasian, 6% African American, and 12.5% of Hispanic ethnicity (United States Census 2010).^36^

This study documents disparity in care limited to a 2006 data set. There was a departmental policy change relating to consultant services providing coverage for facial injuries after 2007 that precluded continuation of the study beyond that point. Similarly, at the same time, there was a transition from scanned paper records to electronic records, which may have impacted the integrity of data during the transition period. Disparities in healthcare is an evolving factor and it is conceivable that there may have been changes since 2006 that could impact outcomes using more recent data. However, despite this limitation, this study brings attention to an important public health issue.

We did not examine whether the repairs performed by a specialty team member were cosmetically comparable to those performed by an ED team member, nor did we investigate the cosmetic impact of procedural sedation. These may be questions for future study. Similarly, it would be interesting to know the degree to which specialty consultation, sedation, or cosmetic factors influenced overall patient and parent satisfaction, although this was not determined in the present study. Nevertheless, prior studies have shown no differences in outcomes or parent satisfaction if laceration repairs are performed by trained nurses when compared to physicians, and other studies have shown that the gender of the physician performing the repair was considered more important by the parents or patients than the level of experience of the physician.^33^–^35^

In many locales, private insurance patients may have greater access than publicly insured patients to urgent care and secondary medical facilities. This could conceivably result in a difference in the types of facial lacerations that present to a tertiary care ED based on patient’s insurance status. However, in the state where the study was conducted, public insurance patients have access to numerous urgent care facilities, and although patients seen in urgent care centers were not included in this study, the Level I PED used for data collection is the major resource in this state for pediatric patients seeking care for facial lacerations, regardless of insurance status, and especially if there is a need for procedural sedation.

## CONCLUSION

Patients with public insurance received less frequent specialty consultation compared to privately insured patients, despite a similarity in the presentation and characteristics of their facial lacerations. While this may not mean that there is a difference in the quality of care provided, future investigation may help to clarify whether this association is the result of caregiver bias, parental expectations, or other unmeasured confounders.

## Figures and Tables

**Table 1:** Patient characteristics and presentation for facial lacerations based on insurance status.

Variable	Private insurance N=667, N (%)	Public insurance N=485, N (%)	p-value
Median age of the patient			
Years [IQR]	4.4 [2.39–7.86]	4.8 [2.80–7.72]	0.18
Gender			
Male	430 (65)	323 (67)	0.45
Female	237 (35)	162 (33)	
Emergency medical service arrival			0.05
No	598 (90)	419 (86)	
Yes	66 (10)	66 (14)	
Missing	3		
Location of injury			
Home	389 (58)	295 (61)	0.58
School	71 (11)	49 (10)	
Street	42 (6)	40 (8)	
Playground/park	27 (4)	21 (4)	
Sports activity (organized)	22 (3)	14 (3)	
Sports activity (unorganized)	16 (2)	13 (3)	
Other	75 (11)	39 (8)	
Missing	25	14	
Mechanism of injury			
Fall	365 (55)	268 (55)	0.6
Struck/against	267 (40)	190 (39)	
Cut/pierce	14 (2)	8 (2)	
Bite/animal/human	14 (2)	9 (2)	
Firearm/gun shot/BB gun/fire	5 (1)	9 (2)	
Missing	2	1	
Intent			
Accidental	645 (97)	464 (96)	0.4
Alleged assault/self-injury/legal	20 (3)	21 (4)	
Missing	2	0	
Acuity level assigned			<0.001
High	25 (4)	16 (3)	
Moderate	259 (39)	126 (26)	
Low	383 (57)	343 (71)	
Length			
≤1cm	324 (49)	237 (49)	0.76
>1cm or ≤2cm	232 (35)	169 (35)	
>2cm	101 (15)	68 (14)	
Missing	10	11	
Depth			0.58
Superficial/simple	321 (48)	243 (50)	
Deep/complicated	295 (44)	212 (44)	
Missing	51	30	

IQR in brackets, N (%) in parentheses.

**Table 2:** Comparison of management of pediatric patients with facial laceration based on insurance status.

Variable	Private insurance N=667, N (%)	Public insurance N=485, N (%)	p-value
Local anesthetic used			0.46
No	200 (30)	162(33)	
Yes	460 (69)	318 (66)	
Missing	7	5	
Local anesthetic used as LET			0.88
No	286 (43)	207 (43)	
Yes	372 (56)	273 (56)	
Missing	9	5	
Local anesthetic used as lidocaine			0.06
No	280 (42)	238 (49)	
Yes	379 (57)	242 (50)	
Missing	8	5	
Specialty consultation obtained			0.0015
No	595 (89)	457 (95)	
Yes	66 (10)	21 (4)	
Missing	6	7	
Procedural sedation used			0.007
No	593 (89)	456 (94)	
Yes	68 (10)	25 (5)	
Missing	6	4	
Physician performing the repair			0.004
PED physician/fellow/resident	599 (90)	461 (95)	
Specialty consult physician	63 (10)	19 (4)	
Missing	5	5	
Median total time in PED			
Minutes [IQR]	180 [124–234]	175 [124–234]	0.11

*LET,* lidocaine, epinephrine, tetracaine mixture; *PED*, pediatric emergency department

IQR ranges in brackets, N (%) in parentheses.

**Table 3:** Multivariate analysis for laceration management.

Variables	Specialty consultation obtainedÞ OR (95% CI)	Procedural sedation use¥ OR (95% CI)
Insurance status		
Private	1.0	1.0
Public	0.45 (0.25–0.78)	0.74 (0.40–1.31)
Self-pay	0.56 (0.18–1.46)	1.54 (0.53–3.86)
Age-years	1.04 (0.98–1.1)	-
Gender		
Male	1.0	-
Female	1.88 (1.15–3.07)	-
Depth		
Superficial/simple	1.0	1.0
Deep/complicated	1.94 (1.13–3.4)	2.47 (1.40–4.44)
Length		
≤1cm	1.0	1.0
>1cm or ≤2cm	0.93 (0.5–1.70)	1.45 (0.79–2.7)
>2cm	1.56 (0.78–3.10)	2.08 (0.98–4.36)
Acuity level assigned		
High level	1.0	1.0
Moderate level	0.27 (0.12–0.64)	0.54 (0.19–1.61)
Low level	0.1 (0.04–0.23)	0.16 (0.06–0.5)
Specialty consultation obtained		
No	-	1.0
Yes	-	17.1 (5.93–50.8)^

Þ Model 1: Consultation obtained=insurance status+age+gender+depth of laceration+length of laceration+acuity.

¥ Model 2: Procedural sedation use=insurance status+age+gender+consult+depth of laceration+length of laceration+acuity+age*consult +gender*consult.

^ Odds ratio for receiving procedural sedation calculated for a two-year old girl.

## References

[1] Kozlowski MJ, Wiater JG, Pasqual RG (2002). Painful discrimination: the differential use of analgesia in isolated lower limb injuries. Am J Emerg Med.

[2] Tamayo-Sarver JH, Hinze SW, Cydulka RK (2003). Racial and ethnic disparities in emergency department analgesic prescription. Am J Public Health.

[3] Slover J, Gibson J, Tosteson T (2005). Racial and economic disparity and the treatment of pediatric fractures. J Pediatr Orthop.

[4] Heron SL, Stettner E, Haley LL (2006). Racial and ethnic disparities in the emergency department: a public health perspective. Emerg Med Clin North Am.

[5] Schrader CD, Lewis LM (2013). Racial disparity in emergency department triage. J Emerg Med.

[6] Sonnenfeld N, Pitts SR, Schappert SM (2012). Emergency department volume and racial and ethnic differences in waiting times in the United States. Med Care.

[7] Wu BU, Banks PA, Conwell DL (2009). Disparities in emergency department wait times for acute gastrointestinal illnesses: results from the National Hospital Ambulatory Medical Care Survey, 1997–2006. Am J Gastroenterol.

[8] Skaggs DL, Lehmann CL, Rice C (2006). Access to orthopaedic care for children with medicaid versus private insurance: results of a national survey. J Pediatr Orthop.

[9] Skinner AC, Mayer ML (2007). Effects of insurance status on children’s access to specialty care: a systematic review of the literature. BMC Health Serv Res.

[10] Svenson JE, Spurlock CW (2001). Insurance status and admission to hospital for head injuries: are we part of a two-tiered medical system?. Am J Emerg Med.

[11] Sabharwal S, Zhao C, McClemens E (2007). Pediatric orthopaedic patients presenting to a university emergency department after visiting another emergency department: demographics and health insurance status. J Pediatr Orthop.

[12] Keith D, Ashby VB, Port FK (2008). Insurance type and minority status associated with large disparities in prelisting dialysis among candidates for kidney transplantation. Clin J Am Soc Nephrol.

[13] Wilper A, Woolhandler S, Himmelstein D (2010). Impact of insurance status on migraine care in the United States: a population-based study. Neurology.

[14] Shi L, Lebrun LA, Tsai J (2010). Access to medical care, dental care, and prescription drugs: the roles of race/ethnicity, health insurance, and income. South Med J.

[15] Cunningham PJ, Tu HT (1997). A changing picture of uncompensated care. Health Aff (Millwood).

[16] Hsia RY, MacIsaac D, Palm E (2008). Trends in charges and payments for nonhospitalized emergency department pediatric visits, 1996–2003. Acad Emerg Med.

[17] Katz MH (2010). Future of the safety net under health reform. JAMA.

[18] Martinez ME, Cohen RA (2012). Health Insurance Coverage: Early Release of Estimates from the National Health Interview Survey.

[19] Rosenbaum S (2008). The proxy war--SCHIP and the government’s role in health care reform. N Engl J Med.

[20] Banthin JS, Bernard DM (2006). Changes in financial burdens for health care: national estimates for the population younger than 65 years, 1996 to 2003. JAMA.

[21] Shi L, Stevens GD (2005). Disparities in access to care and satisfaction among U.S. children: the roles of race/ethnicity and poverty status. Public Health Rep.

[22] Simon HK, Hirsh DA, Rogers AJ (2009). Pediatric Emergency Department Overcrowding: Electronic Medical Record for Identification of Frequent, Lower Acuity Visitors. Can We Effectively Identify Patients for Enhanced Resource Utilization?. J Emerg Med.

[23] Berman S, Armon C, Todd J (2005). Impact of a decline in Colorado Medicaid managed care enrollment on access and quality of preventive primary care services. Pediatrics.

[24] Peterson TH, Peterson T, Armon C (2011). Insurance-associated disparities in hospitalization outcomes of michigan children. J Pediatr.

[25] Knapp JF (1999). Updates in wound management for the pediatrician. Pediatr Clin North Am.

[26] Maizlish N, Herrera L (2006). Race/ethnicity in medical charts and administrative databases of patients served by community health centers. Ethn Dis.

[27] Kressin NR, Chang BH, Hendricks A (2003). Agreement between administrative data and patients’ self-reports of race/ethnicity. Am J Public Health.

[28] U.S. Dept. of Health and Human Services, Centers for Disease Control and Prevention, National Center for Injury Prevention and Control, and United States Consumer Product Safety Commission (2005). National Electronic Injury Surveillance System All Injury Program.

[29] http://www.ahrq.gov/professionals/systems/hospital/esi/index.html.

[30] R Development Core Team (2015). R: A language and environment for statistical computing.

[31] Brodzinski H, Iyer S, Grupp-Phelan J (2010). Assessment of disparities in the use of anxiolysis and sedation among children undergoing laceration repair. Acad Pediatr.

[32] Sanders LM, Thompson VT, Wilkinson JD (2007). Caregiver health literacy and the use of child health services. Pediatrics.

[33] Bonadio WA, Carney M, Gustafson D (1994). Efficacy of nurses suturing pediatric dermal lacerations in an emergency department. Ann Emerg Med.

[34] Charles A, Le Vasseur SA, Castle C (1999). Suturing of minor lacerations by clinical nurse specialists in the emergency department. Accid Emerg Nurs.

[35] Waseem M, Ryan M (2005). “Doctor” or “doctora”: do patients care?. Pediatr Emerg Care.

[36] http://factfinder2.census.gov/faces/tableservices/jsf/pages/productview.xhtml?pid=ACS_12_5YR_S0501.

